# Oral Melatonin Versus Midazolam as Premedication for Intravenous Sedation in Pediatric Dental Patients

**Published:** 2018-09

**Authors:** Ghassem Ansari, Mahnaz Fathi, Masoud Fallahinejad Ghajari, Majid Bargrizan, Ahmad Eghbali

**Affiliations:** 1 Professor, Department of Pediatric Dentistry, Dental School, Shahid Beheshti University of Medical Sciences, Tehran, Iran; 2 Research Institute of Dental Sciences, Dental School, Shahid Beheshti University of Medical Sciences, Tehran, Iran; 3 Fellow Graduate, Department of Pediatric Dentistry, Dental School, Shahid Beheshti University of Medical Sciences, Tehran, Iran; 4 Associate Professor, Department of Pediatric Dentistry, Dental School, Shahid Beheshti University of Medical Sciences, Tehran, Iran; 5 Assistant Professor, Department of Anesthesiology, Mofid Children Hospital, Medical School, Shahid Beheshti University of Medical Sciences, Tehran, Iran

**Keywords:** Premedication, Midazolam, Melatonin, Conscious Sedation

## Abstract

**Objectives:**

This study aimed to evaluate the effect of oral melatonin and oral midazolam as premedication for intravenous (IV) sedation of pediatric dental patients.

**Materials and Methods:**

This crossover, double-blind randomized clinical trial was conducted on 23 uncooperative 2–6-year-olds with definitely negative behaviors according to the Frankl's scale. Each child served as their own control. The children were randomly divided into two groups: group I received 0.5mg/kg of oral melatonin one hour before IV sedation, while group II received 0.5mg/kg of oral midazolam 30 minutes before IV sedation on their first visit. Every child received the other premedication on their second visit. The degree of sedation was judged according to the Houpt scale. Physiologic parameters including blood pressure (PB), heart rate (HR), and blood oxygen saturation (SpO2) and side effects including dizziness, nausea, vomiting, and sleepiness were assessed. The parents' and the operator's satisfaction rates were scored. Data were analyzed using paired t-test and Wilcoxon signed-rank test.

**Results:**

There were significant differences in sedation scores between the two sessions (P<0.05). However, there were no significant differences in alterations of physiologic parameters between the two sessions (P>0.05). Nausea and vomiting were more common during the first two hours in the midazolam group (P=0.002). Tremors were more common in the melatonin group (P=0.013). Dizziness was more evident when melatonin was used (P<0.001). The clinician and the parents were more satisfied with the results of midazolam intake (P<0.05).

**Conclusions:**

Premedication with oral midazolam in pediatric patients is superior to that with melatonin with a higher parents' and operator's satisfaction.

## INTRODUCTION

For many years, pediatric dental treatments have been carried out using conventional behavioral management techniques following the establishment of a good relationship between the dentist and the child as well as the parent(s); this is best achieved in the presence of an efficient local anesthesia [1,2]. However, some children may avoid dental treatment and injection due to fear, low age, and unpleasant previous experience. Earlier studies indicated that high levels of stress and pain before and during dental procedures can significantly affect the level of postoperative pain [3]. Stressful sources such as sounds and smells of dental devices and materials are partly responsible for children’s dental anxiety and poor cooperation [3,4].

Conscious sedation and general anesthesia are two main methods of pharmaceutical interventions [5]. A decrease is obtained in the level of anxiety with an increase of the pain threshold following sedation relative to its depth [6]. Evidence shows that 20% to 50% of children show unfavorable behavioral changes following the first hospital admission [7]. Conscious sedation enables easy and efficient high-quality dental care while controlling children’s behavior and creating a positive attitude towards dental care [8]. Intravenous (IV) sedation is known as the most efficient and safe method when appropriately monitored for patient’s vital signs [9]. Benzodiazepines are the most common sedative medications prescribed for dental anxiety control. Benzodiazepines have been proved to have sedative, hypnotic, and anxiolytic effects when used as premedication in pediatric dentistry [10].

Oral midazolam elixir is one of the most commonly available forms of midazolam prescribed as premedication to overcome behavioral complications at the dental office [11]. However, some degrees of paradoxical reactions and psychomotor disorders have been reported as a consequence of midazolam in few cases [12]. Melatonin is a human physiological hormone produced by the pineal gland, which regulates sleep cycles. Moreover, it is synthesized and available in the form of tablet prescribed for anxiety, pain, and inflammation; it is also a potential replacement for benzodiazepines for premedication [13,14]. Melatonin has a much lower sleep disturbance rate with no postoperative complication when compared to midazolam. Oral administration of 1–5 mg of melatonin elevates its plasma level to 10 to 100 times higher than the endogenous level of melatonin with no side effects such as drowsiness, headache, rash, or insomnia [15]. The efficacy of melatonin for premedication in children has not been well investigated, and limited evidence is available on its routine sleep inducing potentials [16–19,20].

The present study was designed to evaluate the efficacy of oral melatonin as premedication in comparison with oral midazolam in a group of uncooperative 2–6-year-old pediatric dental patients prior to IV ketamine dental sedation in addition to their effect on postoperative side effects of the main sedative drugs.

## MATERIALS AND METHODS

This crossover, double-blind randomized clinical trial was conducted on a group of 2–6-year-old uncooperative children. Ethical approval was obtained from the Ethics Committee of the School of Dentistry of Shahid Beheshti University of Medical Sciences (IR.SBMU.RIDS.REC.1395.209). The study is also registered under IRCT No. IRCT2016101616106N2 as a randomized clinical trial.

Written informed consent was sought from the parents or legal guardians of children during their introductory session. The inclusion criteria included children with definitely negative behaviors according to the Frankl’s behavior rating scale [21], approved by two pediatric dentists, and children classified as ASA I (American Society of Anesthesiologists) with at least two similar teeth requiring similar dental treatments (pulpotomy, restoration, or extraction). The exclusion criteria comprised any systemic medical condition, common cold symptoms including fever (checked using a thermometer; Saadat Monitoring device, Tehran, Iran), cough, nasal discharge, nasal obstruction, respiratory infection, limited neck movement, macroglossia, tonsillar hypertrophy, allergy to medications, micrognathia, limitation in mouth opening, and inability to remain NPO (nothing by mouth) examined by the anesthesiologist in charge. Patients were selected using convenience sampling based on the inclusion and exclusion criteria as well as availability. The sample size was calculated as 44 samples (22 in each group) using Minitab software (Minitab Inc., State College, PA, USA) assuming a=0.05 and b=0.2. The difference in the degree of sedation between the groups was checked according to the Houpt sedation rating scale [22].

The children were randomly assigned to two groups using sequence random allocation by the technician administering the anesthetic with group I receiving 0.5mg/kg of oral melatonin tablets (Vegetarian Formula, Vitane Pharmaceuticals Pvt. Ltd., NY, USA) dissolved in sweetened water as oral premedication to IV sedation on their first visit, while group II received 0.5mg/kg of oral midazolam (Midamax®, Tehran Chemie Pharmaceutical Co., Tehran, Iran) as oral premedication to IV sedation on their first visit. Each child received the other premedication on their second visit; this was to overcome the sequence effect. The patients, the parents, and the pediatric dentists in charge of the assessments were blind to the medications given to the two groups. All the cases had initial records of vital signs including heart rate (HR), respiratory rate (RR), blood pressure (BP), and blood oxygen saturation (SpO2) in addition to these records taken every 15 minutes to the end of the treatment session.

The patients were asked to be NPO for 6 hours prior to each sedation session as a critical requirement of the procedure. Sedation instruction leaflets were given to the parents on the introductory session and prior to the treatment session. The recorded physiologic parameters included HR, SpO2, and BP. The Houpt sedation scale was adopted in order to assess the sedative effect of the premedication at the IV administration stage (Table 1).

**Table 1.** Houpt sedation rating scale

** Parameter **	** Definition **	** Score **
** Sleepiness **	Fully awake, alert	1
	Drowsy, disoriented	2
	Asleep	3

** Movement **	Violent movement that interrupts treatment	1
	Continuous movement that makes treatment difficult	2
	Controllable movement that does not interfere with treatment	3
	No movement	4

** Crying **	Hysterical crying that interrupts treatment	1
	Continuous persistent crying that makes treatment difficult	2
	Intermittent, mild crying that does not interfere with treatment	3
	No crying	4

** Overall behavior **	Aborted: No treatment rendered	1
	Poor: Treatment interrupted, only partial treatment completed	2
	Fair: Treatment interrupted, but eventually all completed	3
	Good: Difficult, but all completed	4
	Very good: Some limited crying or movement, e.g. during anesthesia or mouth prop insertion	5
	Excellent: No crying or movement	6

Sedation assessment was conducted 30 minutes after the premedication and when the patients were expected to receive IV sedative agents including 1–2 mg/kg of ketamine hydrochloride (Rotexmedica, Trittau, Germany), 0.02 mg/kg of atropine (Alborz Darou Pharmaceutical Co., Tehran, Iran), and 0.2 mg/kg of midazolam (Midamax®, Tehran Chemie Pharmaceutical Co., Tehran, Iran). A BP cuff was placed over the left upper arm, and a pulse oximeter probe (Alborz B9, Pooyandegan Rah Saadat Medical Supply Co., Tehran, Iran) was fitted on the index finger of the child’s right hand. Vital signs (physiologic parameters) were recorded as well as the response rate to the administered sedatives according to the Houpt sedation rating scale [22] (Table 1).

Two calibrated, independent pediatric dentists who were blind to the groups’ allocation performed the Houpt sedation rating. Continued monitoring was carried out for the children until full recovery. The parents were contacted by phone 6 and 24 hours after discharge in order to check the postoperative status. Complications and side effects such as drowsiness, dizziness, nausea, and vomiting were recorded as well as the overall satisfaction with the procedure and treatment session. The second appointment was then scheduled for the patients one to two weeks later for the next tooth with a similar treatment need to administer the other premedication to allow for a comparison. Data analysis was carried out using Wilcoxon signed-rank test and paired t-test in SPSS 21 software (IBM Co., Chicago, IL, USA) with P<0.05 as the level of significance.

## RESULTS

All the 23 children involved in this study were judged as definitely negative according to the Frankl's behavior rating scale [21]. The mean age of the patients, including 17 boys (73.9%) and 6 girls (26.1%), was 4±0.9 years, and their mean weight was 16.12±2.58 kg. The most frequent sleepiness score was score 2 (drowsy and disoriented) in the midazolam group (78.3%), while score 1 (fully awake and alert) was the most frequent score (47.8%) in the melatonin group (Table 2).

**Table 2.** Sleepiness scores according to the Houpt sedation rating scale in midazolam and melatonin groups N(%)

** Score **	** 1 **	** 2 **	** 3 **

** Group **			
** Midazolam **	1(4.3)	18(78.3)	4(17.4)
** Melatonin **	11(47.8)	7(30.4)	5(21.7)

Comparison of the two groups showed significant differences in sleepiness with the midazolam group showing higher scores (P=0.011). The most frequent movement score was score 3 (controllable, no interference with treatment) in both groups (82.6% in the midazolam group and 60.9% in the melatonin group; Table 3).

**Table 3.** Movement scores according to the Houpt sedation rating scale in midazolam and melatonin groups N(%)

** Score **	** 1 **	** 2 **	** 3 **	** 4 **

** Group **				
** Midazolam **	0	0	19 (82.6)	4 (17.4)
** Melatonin **	0	8 (34.8)	14 (60.9)	1 (4.3)

Comparison of the two groups showed a significant difference with regard to movement scores (P=0.005). The crying score was also recorded with score 3 (intermittent, mild crying that does not interfere with treatment) as the most frequent score in both groups (73.9% in the midazolam group and 65.2% in the melatonin group; Table 4).

**Table 4.** Crying scores according to the Houpt sedation rating scale in midazolam and melatonin groups N(%)

** Score **	** 1 **	** 2 **	** 3 **	** 4 **

** Group **				
** Midazolam **	0	1 (4.3)	17 (73.9)	5 (21.7)
** Melatonin **	2 (8.7)	4 (17.4)	15 (65.2)	2 (8.7)

A significant difference was evident between the groups with a higher frequency of movement (score 3) in the midazolam group (P=0.021). The overall behavior was scored 5 (47.8%; very good: limited crying or movement) in the midazolam group and 4 (60.9%; good: difficult but all completed) in the melatonin group (Table 5). The overall behavior score was proved significantly different between the two studied groups (P=0.002).

**Table 5.** Overall behavior scores according to the Houpt sedation rating scale in midazolam and melatonin groups N(%)

** Score **	** 1 **	** 2 **	** 3 **	** 4 **	** 5 **	** 6 **

** Group **						
** Midazolam **	0	0	2(8.7)	6(26.1)	11(47.8)	4(17.4)
** Melatonin **	2(8.7)	0	5(21.7)	14(60.9)	1(4.3)	1(4.3)

Common postoperative complications included nausea, vomiting, tremors, and dizziness during the first 24 hours reported by the parents. Hallucination was not reported in any of the treated cases. Nausea and vomiting were reported in 10 patients of the midazolam group during the first two hours after treatment; the difference between the groups was statistically significant (P=0.002; Table 6).

**Table 6.** Frequency of postoperative complications in midazolam and melatonin groups

** Group **		** Midazolam N(%) **	** Melatonin N(%) **	** P-value **

** Complications **				
** Nausea and vomiting **	First 2 hours	10(43.5)	0	P=0.002
	First 6 hours	1(4.3)	0	
	First 6-24 hours	0	0	

** Tremors **	Presence	11(47.8)	2(8.7)	P=0.013
	Absence	12(52.2)	21(91.3)	

** Dizziness **	Presence	16(69.6)	3(13)	P<0.001
	Absence	7(30.4)	20(87)	

** Hallucination **	Presence	0	0	-
	Absence	23(100)	23(100)	

Only one patient was reported as experiencing some degrees of nausea 6 hours following treatment in the midazolam group. Tremors were noted in 11 (47.8%) patients in the midazolam group and in 2 (8.7%) patients in the melatonin groups with statistically significant differences (P=0.013). Dizziness was noted in 16 (69.6%) patients in the midazolam group and in only 3 (13%) patients in the melatonin group with the difference being statistically significant (P<0.001).

The clinician’s judgment indicated that the patients of the midazolam group showed acceptable levels of sedation in 65.2% of the cases, while the melatonin group showed acceptable levels of sedation in 26.1% of the cases; the difference was statically significant (P<0.05). The parents’ satisfaction assessment using a postoperative interview was indicative of 69.6% effectiveness of midazolam; this rate was 30.4% for melatonin with the difference being statistically significant (P<0.05). The pattern of HR changes was not different between the two groups, whereas the HR increased when treatment time was prolonged (P<0.001; Table 6). The difference in the HR between the two groups was not significant at any time point (P=0.71). The RR was almost the same in the two groups (P=0.0671) with the changes in the RR showing no significant difference between the groups (P=0.598).

No change in the SpO2 was evident in the melatonin group, while the midazolam group showed some degrees of change at the time of IV administration of sedatives (98.30±1.82%), and the SpO2 was lowest upon the completion of treatment (93.45±3.06%). The difference in the SpO2 rate between the two groups was not significant overall (P=0.890) or at any time point (P=0.322 at the baseline and 0.861 at IV administration). The BP was almost the same in the two groups (P=0.347), and the intragroup changes were not significant (P=0.340).

## DISCUSSION

Evidence shows that high levels of anxiety could prevent many fearful young and adult patients from presenting to dentists for their dental treatment needs [23]. Various types and levels of premedication have been recommended to eliminate the initial anxiety of such children prior to dental procedures. Benzodiazepines are considered as the drug of choice for premedication [24]. Among all types of benzodiazepine, midazolam is known as a very safe and common premedication in children [11]. It is of note that sporadic cases have been reported on some known potential side effects such as paradoxical effects, interference with opioids, excessive drowsiness, psychomotor disorders, and amnesia [11]. In an attempt to find a natural alternative to midazolam with the same level of efficacy, this study compared the sedative efficacy as well as side effects of melatonin and midazolam in pediatric dental patients. The results indicated that although the Houpt scores recorded were relatively higher in the midazolam group, the overall potential side effects were almost none in the melatonin group, while some side effects were noticed in the midazolam group. Physiological parameters were not significantly different between the two groups. Both the clinician’s and the parent’s satisfaction rates were significantly more in favor of the effects of midazolam.

Melatonin is a hormone famous for its anxiolytic, analgesic, and anti-inflammatory properties [25]. It has been used as a premedication in children requiring sedation or general anesthesia for diagnostic or therapeutic procedures with favorable results [17–19]. Optimal efficacy of melatonin has been documented as an initial anxiolytic agent in pediatric patients scheduled for surgical operations [26]. Moreover, the risk of anxiety and sleep disturbances is much lower following the use of melatonin compared to midazolam, with further preventive postoperative delirium effect [27]. Melatonin and its analogs have been suggested as an alternative to midazolam for premedication in children [28]. However, its efficacy and adequate dosage in children are yet to be defined; the optimal dose of 0.5 mg/kg was adopted for melatonin based on earlier reports [29].

Dental sedation is best performed by the use of more natural products such as human natural hormones. Melatonin with its advocated sedative effects was used in the present study to compare its efficacy to currently popular midazolam for sedation.

Acil et al [30] reported that premedication with melatonin has preoperative anxiolytic and sedative effects without postoperative psychomotor impairment. Ionescu et al [31] concluded that 3 mg of melatonin can be successfully used as premedication for its optimal anxiolytic and analgesic properties and faster recovery. Samarkandi et al [32] reported that premedication with 0.1, 0.25, and 0.5 mg/kg of melatonin is as effective as that with 0.1, 0.25, and 0.5 mg/kg of midazolam for the management of anxiety in children. Perez-Heredia et al [33] and Kain et al [34] compared the effect of 0.5 mg/kg of midazolam with that of 0.4–0.5 mg/kg of melatonin as premedication in order to control anxiety in children with the superiority of midazolam to melatonin in the reduction of anxiety. According to Seet et al [35], melatonin is believed to act more efficiently in females in controlling pain and anxiety as a natural hormone. Isik et al [16] compared 3 mg of melatonin with 0.75 mg/kg of midazolam and a placebo as premedication in children requiring sedation for dental treatments and reported that melatonin was similar to the placebo. Sury and Fairweather [19] reported that melatonin was not effective enough when compared to Temazepam and Droperidol before magnetic resonance imaging (MRI).

In the present research, all the parameters of the Houpt sedation rating scale were found to show higher rates in the midazolam group with sleepiness, crying, movement, and the overall behavior all being significantly different when compared to those related to melatonin. Midazolam and melatonin were not significantly different in terms of changes in the HR, RR, or SpO2 at different time points recorded. Melatonin was employed in order to avoid the possibility of a synergistic effect from various premedication combinations used as sedatives in addition to the elimination of any misinterpretation. Difficulty in recruiting cooperative patients was considered as a limitation. Unavailability of a standard form of oral melatonin suspension for children could be referred to as another limitation; therefore, the tablet form was ground and dissolved in water for easier oral intake. It is recommended to choose a larger sample size in order to further assess the outcome as even the recent reports on melatonin have little to add to what this article has to offer [33,35].

## CONCLUSION

Within the limitations of this study, it appears that premedication with 0.5 mg/kg of midazolam is superior to premedication with 0.5 mg/kg of melatonin when compared in terms of the sedative efficacy. A higher frequency of side effects such as dizziness and nausea is associated with the use of midazolam, while there are no such side effects with melatonin.
